# A Method to Dynamic Stochastic Multicriteria Decision Making with Log-Normally Distributed Random Variables

**DOI:** 10.1155/2013/202085

**Published:** 2013-09-26

**Authors:** Xin-Fan Wang, Jian-Qiang Wang, Sheng-Yue Deng

**Affiliations:** ^1^School of Science, Hunan University of Technology, Zhuzhou 412007, China; ^2^School of Business, Central South University, Changsha 410083, China

## Abstract

We investigate the dynamic stochastic multicriteria decision making (SMCDM) problems, in which the criterion values take the form of log-normally distributed random variables, and the argument information is collected from different periods. We propose two new geometric aggregation operators, such as the log-normal distribution weighted geometric (LNDWG) operator and the dynamic log-normal distribution weighted geometric (DLNDWG) operator, and develop a method for dynamic SMCDM with log-normally distributed random variables. This method uses the DLNDWG operator and the LNDWG operator to aggregate the log-normally distributed criterion values, utilizes the entropy model of Shannon to generate the time weight vector, and utilizes the expectation values and variances of log-normal distributions to rank the alternatives and select the best one. Finally, an example is given to illustrate the feasibility and effectiveness of this developed method.

## 1. Introduction 

In the socioeconomic activities, there are a large number of stochastic multicriteria decision making (SMCDM) problems in which the criterion values take the form of random variables [1–13]. In SMCDM problems, the normal distribution with well-known bell-shaped curve is most often assumed to describe the random variation that occurs in the criterion values, and each criterion value is commonly characterized and described by two values: the arithmetic mean and the standard deviation [1, 14, 15]. 

However, many measurements of criterion values show a more or less skewed distribution. Particularly, skewed distributions are common when mean values are low, variances large, and values cannot be negative. Such skewed distributions often approximately fit the log-normal distribution [16, 17]. The log-normal distribution is a continuous probability distribution of a random variable whose logarithm is normally distributed [16, 18]. It is similar to the normal distribution, but there are still several major differences between them: first, the normal distribution is symmetrical; the log-normal distribution is skewed to the left. Second, both forms of normal and log-normal variability are based on a variety of forces acting independent of one another, but a major difference is that the effects can be additive or multiplicative, thus leading to normal or log-normal distributions, respectively. A variable might be distributed as log-normally if it can be thought of as the multiplicative product of a large number of independent random variables each of which is positive. Third, the sum of several independent normal distributed random variables itself is a normal distributed random variable. For log-normally distributed random variables, however, multiplication is the relevant operation for combining them in most applications; that is, the product of several independent log-normal random variables also follows a log-normal distribution. The log-normal distribution can model many instances, such as the loss of investment risk, the change in price distribution of a stock, and the failure rates in product tests [16, 19–21]. This is because the time series creates random variables. By taking the natural log of each of the random variables, the resulting set of numbers shall be distributed log-normally. Thus, in real-life, there are many SMCDM problems in which the criterion values take the form of log-normally distributed random variables.

At present, the SMCDM problems, in which the criterion values take the form of normally distributed random variables, have attracted lots of attentions from researchers [1–8]. But regarding the SMCDM problems, in which the criterion values take the form of log-normally distributed random variables, there is still few related research.

Moreover, in some SMCDM situations, such as multi-periods investment decision making, medical dynamic diagnosis, personnel dynamic examination, military system efficiency dynamic evaluation, etc., the original decision information may be collected at different periods (for convenience, we call this kind of SMCDM problems the dynamic SMCDM problems) [8]. Thus, accordingly, time should be taken into account, and it is an interesting and important research issue. 

In this paper, we shall focus on the dynamic SMCDM problems, in which the criterion values take the form of log-normally distributed random variables and the argument information is given at different periods, and develop a method for dynamic SMCDM with log-normally distributed random variables. This method uses two new geometric aggregation operators to aggregate the log-normally distributed criterion values, utilizes the entropy model of Shannon to generate the time weight vector, and utilizes the expectation values and variances of log-normal distributions to rank the alternatives and select the best one.

To do so, this paper is organized as follows.  Section 2 introduces some operational laws of log-normal distributions and presents a method for the comparison between two log-normal distributions.  Section 3 proposes two new geometric aggregation operators, such as the log-normal distribution weighted geometric (LNDWG) operator and the dynamic log-normal distribution weighted geometric (DLNDWG) operator.  Section 4 develops an approach to solve the dynamic SMCDM problems, in which the criterion values take the form of log-normally distributed random variables, and the argument information is given at different periods.  Section 5 gives an illustrative example. Finally, we conclude the paper in Section 6.

## 2. Preliminaries

The normal distribution is a continuous probability distribution defined by the following probability density function [22]:

(1) $$\begin{array}{c} f_{X}\left(x\right)=\frac{1}{\sqrt{2\pi }\sigma }e^{-{\left(x-\mu \right)}^{2}/2\sigma^{2}},\;-\infty <x<+\infty , \end{array}$$

where *μ* is the expectation, *σ* > 0 is the standard deviation, and *σ* ^2^ is the variance. Generally, we use *X* ~ *N*(*μ*, *σ* ^2^) as a mathematical expression meaning that *X* is distributed normally with the expectation *μ* and variance *σ* ^2^.

The log-normal distribution is a probability distribution of a random variable whose logarithm is normally distributed [16]; that is, if ln⁡*Y* ~ *N*(*μ*, *σ* ^2^), then *Y* has a log-normal distribution. The probability density function of the log-normal distribution has the following form:

(2) $$\begin{array}{c} f_{Y}\left(y\right)=\frac{1}{y\sigma \sqrt{2\pi }}e^{-{\left(\ln y-\mu \right)}^{2}/2\sigma^{2}},\;y>0. \end{array}$$

If *Y* is distributed log-normally with parameters *μ* and *σ*, then we write *Y* ~ log⁡−*N*(*μ*, *σ* ^2^), and for convenience, we call *β* = log⁡−*N*(*μ*, *σ* ^2^) a log-normal distribution, and let Θ be the set of all log-normal distributions.

**Definition 1 (see [16])**

Let *β*_1_ = log⁡−*N*(*μ*_1_, *σ*_1_^2^) and *β*_2_ = log⁡−*N*(*μ*_2_, *σ*_2_^2^) be two log-normal distributions, then

1. *β*_1_ ⊗ *β*_2_ = log⁡−*N*(*μ*_1_ + *μ*_2_, *σ*_1_^2^ + *σ*_2_^2^);
2. *β*_1_^*a*^ = log⁡−*N*(*aμ*_1_, *a* ^2^ *σ*_1_^2^), *a* ≠ 0.

It is easy to prove that all operational results are still log-normal distributions, and by these two operational laws, we have

1. *β*_1_ ⊗ *β*_2_ = *β*_2_ ⊗ *β*_1_;
2. (*β*_1_ ⊗ *β*_2_) ⊗ *β*_3_ = *β*_1_ ⊗ (*β*_2_ ⊗ *β*_3_);
3. (*β*_1_ ⊗ *β*_2_)^*a*^ = *β*_1_^*a*^ ⊗ *β*_2_^*a*^, *a* ≠ 0;
4. *β*_1_^*a*_1_^ ⊗ *β*_1_^*a*_2_^ = *β*_1_^*a*_1_+*a*_2_^, *a*_1_, *a*_2_ ≠ 0.

Furthermore, if log⁡−*N*(*μ*, *σ* ^2^) is a log-normal distribution, then its expected value *μ*_log⁡_ and standard deviation *σ*_log⁡_ can be calculated by the following formulas [16]:

(3) $$\begin{array}{c} \mu_{\log}=e^{\mu +(1/2)\sigma^{2}},\\ \sigma_{\log}=e^{\mu +(1/2)\sigma^{2}}\sqrt{e^{\sigma^{2}}-1}. \end{array}$$

According to the relation between expectation and variance in statistics, in the following, we propose a method for the comparison between two log-normal distributions, which is based on the expected value *μ*_log⁡_ and the standard deviation *σ*_log⁡_.

**Definition 2**

Let *β*_1_ = log⁡−*N*(*μ*_1_, *σ*_1_^2^) and *β*_2_ = log⁡−*N*(*μ*_2_, *σ*_2_^2^) be two log-normal distributions, then

1. if *μ*_log⁡_(*β*_1_) < *μ*_log⁡_(*β*_2_), then *β*_1_ is smaller than *β*_2_, denoted by *β*_1_ < *β*_2_;
2. if *μ*_log⁡_(*β*_1_) = *μ*_log⁡_(*β*_2_), then
  - if *σ*_log⁡_(*β*_1_) = *σ*_log⁡_(*β*_2_), then *β*_1_ is equal to *β*_2_, denoted by *β*_1_ = *β*_2_;
  - if *σ*_log⁡_(*β*_1_) < *σ*_log⁡_(*β*_2_), then *β*_1_ is bigger than*β*_2_, denoted by *β*_1_ > *β*_2_;
  - if *σ*_log⁡_(*β*_1_) > *σ*_log⁡_(*β*_2_), then *β*_1_ is smaller than *β*_2_, denoted by *β*_1_ < *β*_2_.

## 3. The LNDWG and DLNDWG Operators

To aggregate the log-normally distributed criterion values, in what follows, based on Definition 1, we first propose a new geometric aggregation operator, which is called the LNDWG operator.

**Definition 3**

Let *β*_*j*_ = log⁡−*N*(*μ*_*j*_, *σ*_*j*_^2^)  (*j* = 1,2,…, *n*) be a collection of log-normal distributions, and let LNDWG Θ^*n*^ → Θ, if

(4) $$\begin{array}{c} {\text{LNDWG}}_{w}\left(\beta_{1},\beta_{2},\ldots ,\beta_{n}\right)=\beta_{1}^{w_{1}}⊗\beta_{2}^{w_{2}}⊗\cdots ⊗\beta_{n}^{w_{n}}, \end{array}$$

then LNDWG is called the log-normal distribution weighted geometric operator of dimension *n*, where *w* = (*w*_1_, *w*_2_,…, *w*_*n*_)^*T*^ is the weight vector of *β*_*j*_  (*j* = 1,2,…, *n*), with *w*_*j*_ ≥ 0 and ∑_*j*=1_^*n*^ *w*_*j*_ = 1.

**Theorem 4**

Let *β*_*j*_ = log⁡−*N*(*μ*_*j*_, *σ*_*j*_^2^)  (*j* = 1,2,…, *n*) be a collection of log-normal distributions, and let *w* = (*w*_1_, *w*_2_,…, *w*_*n*_)^*T*^ be the weight vector of *β*_*j*_  (*j* = 1,2,…, *n*), with *w*_*j*_ ≥ 0 and ∑_*j*=1_^*n*^ *w*_*j*_ = 1; then their aggregated result using the LNDWG operator is also a log-normal distribution, and

(5) $$\begin{array}{c} {\text{LNDWG}}_{w}\left(\beta_{1},\beta_{2},\ldots ,\beta_{n}\right)=\log-N\left(\overset{n}{\underset{j=1}{\sum }}w_{j}\mu_{j},\overset{n}{\underset{j=1}{\sum }}w_{j}^{2}\sigma_{j}^{2}\right). \end{array}$$

**Proof**

Obviously, from Definition 1, the aggregated value by using the LNDWG operator is also a log-normal distribution. In the following, we prove (5) by using mathematical induction on *n*.

(1) For *n* = 2, since
  (6) $$\begin{array}{c} \beta_{1}^{w_{1}}=\log-N\left(w_{1}\mu_{1},w_{1}^{2}\sigma_{1}^{2}\right),\\ \beta_{2}^{w_{2}}=\log-N\left(w_{2}\mu_{2},w_{2}^{2}\sigma_{2}^{2}\right), \end{array}$$

then

(7) LNDWGw(β1,β2)=β1w1⊗β2w2=log⁡−N(w1μ1+w2μ2,w12σ12+w22σ22)=log⁡−N(∑j=12wjμj,∑j=12wj2σj2).

(2) If (5) holds for *n* = *k*, that is,
  (8) $$\begin{array}{c} {\text{LNDWG}}_{w}\left(\beta_{1},\beta_{2},\ldots ,\beta_{k}\right)=\log-N\left(\overset{k}{\underset{j=1}{\sum }}w_{j}\mu_{j},\overset{k}{\underset{j=1}{\sum }}w_{j}^{2}\sigma_{j}^{2}\right). \end{array}$$

Then, when *n* = *k* + 1, by Definition 1, we have

(9) LNDWGw(β1,β2,…,βk,βk+1)  =log⁡−N(∑j=1kwjμj,∑j=1kwj2σj2)   ⊗(log⁡−N(μk+1,σk+12))wk+1  =log⁡−N(∑j=1kwjμj,∑j=1kwj2σj2)   ⊗log⁡−N(wk+1μk+1,wk+12σk+12)  =log⁡−N(∑j=1kwjμj+wk+1μk+1,∑j=1kwj2σj2+wk+12σk+12)  =log⁡−N(∑j=1k+1wjμj,∑j=1k+1wj2σj2).

That is, (5) holds for *n* = *k* + 1.

Thus, based on (1) and (2), (5) holds for all *n* ∈ *N*, which completes the proof of Theorem 4.

The LNDWG operator is an extension of the well-known weighted geometric averaging (WGA) operator [23]. Similar to the WGA operator, the LNDWG operator has the following properties.

**Theorem 5 (properties of LNDWG)**

Let *β*_*j*_ = log⁡−*N*(*μ*_*j*_, *σ*_*j*_^2^)  (*j* = 1,2,…, *n*) be a collection of log-normal distributions, and let *w* = (*w*_1_, *w*_2_,…, *w*_*n*_)^*T*^ be the weight vector of *β*_*j*_  (*j* = 1,2,…, *n*), with *w*_*j*_ ∈ [0,1] and ∑_*j*=1_^*n*^ *w*_*j*_ = 1; then we have the following.

(1) Idempotency: If all *β*_*j*_  (*j* = 1,2,…, *n*) are equal, that is, *β*_*j*_ = *β* for all *j*, then
  (10) $$\begin{array}{c} {\text{LNDWG}}_{w}\left(\beta_{1},\beta_{2},\ldots ,\beta_{n}\right)=\beta . \end{array}$$
(2) Boundary: min⁡(*β*_1_, *β*_2_,…, *β*_*n*_) ≤ LNDWG_*w*_(*β*_1_, *β*_2_,…, *β*_*n*_) ≤ max⁡(*β*_1_, *β*_2_,…, *β*_*n*_).
(3) Monotonicity: Let *β*_*j*_ = log⁡−*N*(*μ*_*j*_, *σ*_*j*_^2^) and *β*_j_* = log⁡−*N*(*μ*_j_*, (*σ*_j_*)^2^)  (*j* = 1,2,…, *n*) be two collections of log-normal distributions. If *β*_*j*_ ≤ *β*_j_*, for all *j*, then
  (11) $$\begin{array}{c} {\text{LNDWG}}_{w}\left(\beta_{1},\beta_{2},\ldots ,\beta_{n}\right)\leq {\text{LNDWG}}_{w}\left(\beta_{1}^{*},\beta_{2}^{*},\ldots ,\beta_{n}^{*}\right). \end{array}$$

Consider that in many SMCDM problems, the original decision information is usually collected at different periods; then the aggregation operator and its associated weights should not keep constant. In the following, based on Definitions 1 and 3, we propose another new aggregation operator for aggregating the log-normally distributed criterion values given at different periods.

**Definition 6**

Let *t* be a time variable and *Y* be a random variable, if *Y* ~ log⁡−*N*(*μ*(*t*), (*σ*(*t*))^2^) at the period *t*, where *μ*(*t*) and (*σ*(*t*))^2^ is the expectation and the variance of *Y* at the period *t*, respectively, then we call log⁡−*N*(*μ*(*t*), (*σ*(*t*))^2^) the log-normal distribution of *Y*  at the period *t*, denoted by *β*(*t*) = log⁡−*N*(*μ*(*t*), (*σ*(*t*))^2^).

Similar to Definitions 1 and 3, we have the following.

**Definition 7**

Let *β*(*t*_1_) = log⁡−*N*(*μ*(*t*_1_), (*σ*(*t*_1_))^2^) and *β*(*t*_2_) = log⁡−*N*(*μ*(*t*_2_), (*σ*(*t*_2_))^2^) be two log-normal distributions at two different periods *t*_1_, *t*_2_, respectively; then their operational laws can be defined as follows:

1. *β*(*t*_1_) ⊗ *β*(*t*_2_) = log⁡−*N*(*μ*(*t*_1_) + *μ*(*t*_2_), (*σ*(*t*_1_))^2^ + (*σ*(*t*_2_))^2^);
2. (*β*(*t*_1_))^*a*^ = log⁡−*N*(*aμ*(*t*_1_), *a* ^2^(*σ*(*t*_1_))^2^), *a* ≠ 0.

**Definition 8**

Let (*t*_*k*_) = log⁡−*N*(*μ*(*t*_*k*_), (*σ*(*t*_*k*_))^2^)  (*k* = 1,2,…, *p*) be a collection of *p* log-normal distributions at *p* different periods *t*_*k*_  (*k* = 1,2,…, *p*), and let *λ*(*t*) = (*λ*(*t*_1_), *λ*(*t*_2_),…, *λ*(*t*_*p*_))^*T*^ be the weight vector of the periods *t*_*k*_  (*k* = 1,2,…, *p*), with *λ*(*t*_*k*_) ≥ 0 and ∑_*k*=1_^*p*^ *λ*(*t*_*k*_) = 1; then we call

(12) DLNDWGλ(t)(β(t1),β(t2),…,β(tp))  =(β(t1))λ(t1)⊗(β(t2))λ(t2)⊗⋯⊗(β(tn))λ(tn)

the dynamic log-normal distribution weighted geometric (DLNDWG) operator.

**Theorem 9**

Let (*t*_*k*_) = log⁡−*N*(*μ*(*t*_*k*_), (*σ*(*t*_*k*_))^2^)  (*k* = 1,2,…, *p*) be a collection of *p* log-normal distributions at *p* different periods *t*_*k*_  (*k* = 1,2,…, *p*), and let *λ*(*t*) = (*λ*(*t*_1_), *λ*(*t*_2_),…, *λ*(*t*_*p*_))^*T*^ be the weight vector of the periods *t*_*k*_  (*k* = 1,2,…, *p*), with *λ*(*t*_*k*_) ≥ 0 and ∑_*k*=1_^*p*^ *λ*(*t*_*k*_) = 1; then their aggregated result using the DLNDWG operator is also a log-normal distribution, and

(13) DLNDWGλ(t)(β(t1),β(t2),…,β(tp))  =log⁡−N(∑k=1pλ(tk)μ(tk),∑k=1p(λ(tk))2(σ(tk))2).

In (12) and (13), the time weight vector *λ*(*t*) reflects the importance degree of different periods, which can be given by decision maker(s) or can be obtained by using one of the existing methods, including the arithmetic series based method [24], the geometric series based method [24], the BUM function based method [25], the normal distribution based method [25], the exponential distribution based method [26], the Poisson distribution based method [27], the binomial distribution based method [28], and the average age method [25]. In the following, we propose another method to generate the time weight vector *λ*(*t*) = (*λ*(*t*_1_), *λ*(*t*_2_),…, *λ*(*t*_*p*_))^*T*^ by using the entropy model of Shannon [29–31]. Consider that, on one hand, the real weights of different periods are random variables and we can utilize the time weight vector's entropy *H*(*λ*(*t*)) to describe the uncertainty of the time weight vector *λ*(*t*) [30], which is defined as

(14) $$\begin{array}{c} H\left(\lambda \left(t\right)\right)=-\overset{p}{\underset{k=1}{\sum }}\lambda \left(t_{k}\right)\ln\lambda \left(t_{k}\right). \end{array}$$

On the other hand, we can associate with a concept of the relative average age of the data [31], which is defined as

(15) $$\begin{array}{c} \tau =\frac{1}{p-1}\overset{p}{\underset{k=1}{\sum }}\left(p-k\right)\lambda \left(t_{k}\right), \end{array}$$

where *τ* indicates the relative average age of the data. 

The concept of relative average age is an extension of the average age concept [25, 31]. The average age of the data is defined by $\overset{-}{t}=\sum_{k=1}^{p}\left(p-k)\lambda (t_{k}\right)$, but $\overset{-}{t}$ only can be obtained by using approximate method. The relative average age *τ* reflects the degree paid attention to the data of different periods by the decision makers in the process of information aggregation and can be represented by using a 0.1–0.9 scale (Table 1). When *τ* is close to 0, it indicates that the decision makers pay more attention to recent data; when *τ* is close to 1, it indicates that the decision makers pay more attention to distant data; when *τ* = 0.5, it indicates that the decision makers pay the same attention to every period, with no preference. Particularly, when *τ* = 1, then *λ*(*t*) = (1,0,…, 0)^*T*^; when *τ* = 0, then *λ*(*t*) = (0,0,…, 1)^*T*^; when *τ* = 0.5, then *λ*(*t*) = (1/*p*, 1/*p*,…, 1/*p*)^*T*^. 

Thus, we can obtain the time weights by maximizing the time weight vector's entropy *H*(*λ*(*t*)) for a specified level of the relative average age *τ* and then find a set of weights that satisfies the following mathematical programming model for the *λ*(*t*_*k*_):

(M-1)  Maximize: H(λ(t))=−∑k=1pλ(tk)ln⁡λ(tk), Subject  to: τ=1p−1∑k=1p(p−k)λ(tk)             ∑k=1pλ(tk)=1       λ(tk)≥0, k=1,2,…,p.

## 4. A Procedure for Dynamic SMCDM with Log-Normally Distributed Random Variables

In this section, we consider a dynamic SMCDM problem where all criterion values take the form of log-normally distributed random variables collected at different periods. The following notations are used to depict the considered problems.

- *A* = {*A*_1_, *A*_2_,…, *A*_*m*_}: a discrete set of *m* feasible alternatives.
- *I* = {*I*_1_, *I*_2_,…, *I*_*n*_}: a finite set of criteria. The criterion weight vector is *w* = (*w*_1_, *w*_2_,…, *w*_*n*_)^*T*^, with *w*_*j*_ ≥ 0 and ∑_*j*=1_^*n*^ *w*_*j*_ = 1.
- There are *p* different periods *t*_*k*_  (*k* = 1,2,…, *p*) with *t*_*p*_ being the most recent period and *t*_1_ being the most distant period.
- *R*(*t*_*k*_) = (*β*_*ij*_(*t*_*k*_))_*m*×*n*_  (*k* = 1,2,…, *p*): *k* log-normal distribution decision matrices at the periods *t*_*k*_  (*k* = 1,2,…, *p*), where *β*_*ij*_(*t*_*k*_) = log⁡−*N*(*μ*_*ij*_(*t*_*k*_), (*σ*_*ij*_(*t*_*k*_))^2^) are the criterion values of the alternatives *A*_*i*_ with respect to the criteria *I*_*j*_ at the periods *t*_*k*_  (*i* = 1,2,…, *m*, *j* = 1,2,…, *n*, *k* = 1,2,…, *p*).

Based on the above decision information, in the following, we develop a practical procedure to rank the alternatives and select the most desirable one.

**Step 1**

Utilize the model ((M-1)) to generate the time weight vector *λ*(*t*) = (*λ*(*t*_1_), *λ*(*t*_2_),…, *λ*(*t*_*p*_))^*T*^.

**Step 2**

Utilize the DLNDWG operator:

(16) $$\begin{array}{c} \beta_{ij}={\text{DLNDWG}}_{\lambda (t)}\left(\beta_{ij}\left(t_{1}\right),\beta_{ij}\left(t_{2}\right),\ldots ,\beta_{ij}\left(t_{p}\right)\right), \end{array}$$

to aggregate all the log-normal distribution decision matrices *R*(*t*_*k*_) = (*β*_*ij*_(*t*_*k*_))_*m*×*n*_  (*k* = 1,2,…, *p*) into an overall log-normal distribution decision matrix *R* = (*β*_*ij*_)_*m*×*n*_ = (log⁡−*N*(*μ*_*ij*_, *σ*_*ij*_^2^))_*m*×*n*_, where

(17) $$\begin{array}{c} \beta_{ij}=\log-N\left(\mu_{ij},\sigma_{ij}^{2}\right),\;\;\mu_{ij}=\overset{p}{\underset{k=1}{\sum }}\lambda \left(t_{k}\right)\mu_{ij}\left(t_{k}\right),\\ \sigma_{ij}^{2}=\overset{p}{\underset{k=1}{\sum }}{\left(\lambda \left(t_{k}\right)\right)}^{2}{\left(\sigma_{ij}\left(t_{k}\right)\right)}^{2},\\ i=1,2,\ldots ,m,\;\;j=1,2,\ldots ,n \end{array}$$

and *λ*(*t*) = (*λ*(*t*_1_), *λ*(*t*_2_),…, *λ*(*t*_*p*_))^*T*^ is the time weight vector, with *λ*(*t*_*k*_) ≥ 0 and ∑_*k*=1_^*p*^ *λ*(*t*_*k*_) = 1.

**Step 3**

Normalize the decision matrix *R* = (*β*_*ij*_)_*m*×*n*_. Let *I* ^*b*^ be the set of all benefit criteria, and let *I* ^*c*^ be the set of all cost criteria; then we can use the following formulas to transform the decision matrix *R* = (*β*_*ij*_)_*m*×*n*_ into the corresponding normalized decision matrix $\tilde{R}={\left({\tilde{\beta }}_{ij}\right)}_{m\times n}=(\log-N({\tilde{\mu }}_{ij},{\tilde{\sigma }}_{ij}^{2}{))}_{m\times n}$:

(18) $$\begin{array}{c} {\tilde{\mu }}_{ij}=\frac{\mu_{ij}}{\max_{i}\left\{\mu_{ij}\right\}},\;I_{j}\in I^{b},\;\;i=1,2,\ldots ,m, \end{array}$$

(19) $$\begin{array}{c} {\tilde{\mu }}_{ij}=\frac{\min_{i}\left\{\mu_{ij}\right\}}{\mu_{ij}},\;I_{j}\in I^{c},\;\;i=1,2,\ldots ,m, \end{array}$$

(20) $$\begin{array}{c} {\tilde{\sigma }}_{ij}=\frac{\sigma_{ij}}{\max_{i}\left\{\mu_{ij}\right\}},\;I_{j}\in I,\;\;i=1,2,\ldots ,m. \end{array}$$

Note that standard deviation is relative to mean, so (20) is suitable for all *I*_*j*_ ∈ *I*.

**Step 4**

Utilize the LNDWG operator

(21) $$\begin{array}{c} {\tilde{\beta }}_{i}={\text{LNDWG}}_{w}\left({\tilde{\beta }}_{i1},{\tilde{\beta }}_{i2},\ldots ,{\tilde{\beta }}_{in}\right), \end{array}$$

to aggregate the overall criterion values ${\tilde{\beta }}_{ij}$ in the *i*th column of the normalized decision matrix $\tilde{R}={\left({\tilde{\beta }}_{ij}\right)}_{m\times n}$ into the complex overall values ${\tilde{\beta }}_{i}$ of the alternatives *A*_*i*_  (*i* = 1,2,…, *m*), where

(22) $$\begin{array}{c} {\tilde{\beta }}_{i}=\log-N\left({\tilde{\mu }}_{i},{{\tilde{\sigma }}_{i}}^{2}\right),\;{\tilde{\mu }}_{i}=\overset{n}{\underset{j=1}{\sum }}w_{j}{\tilde{\mu }}_{ij}\\ {\tilde{\sigma }}_{i}^{2}=\overset{n}{\underset{j=1}{\sum }}w_{j}^{2}{\tilde{\sigma }}_{ij}^{2},\;i=1,2,\ldots ,m. \end{array}$$

**Step 5**

Utilize (3) to calculate the expected values $\mu_{\log}({\tilde{\beta }}_{i})$ and the standard deviations $\sigma_{\log}({\tilde{\beta }}_{i})$ of the complex overall values ${\tilde{\beta }}_{i}$ of the alternatives *A*_*i*_  (*i* = 1,2,…, *m*).

**Step 6**

Use Definition 2 to rank all the alternatives *A*_*i*_  (*i* = 1,2,…, *m*) and then select the best one according to the values $\mu_{\log}({\tilde{\beta }}_{i})$ and $\sigma_{\log}\left({\tilde{\beta }}_{i}\right)\;\;(i=1,2,\ldots ,m)$.

## 5. Illustrative Example

In this section, we use a practical dynamic SMCDM problem (adapted from [2]) to illustrate the application of the developed approach.

An investment company wants to invest a total amount of money in the best option. There are five possible companies *A*_*i*_  (*i* = 1,2,…, 5) to be invested: (1)  *A*_1_ is an arms company; (2)  *A*_2_ is a computer company; (3)  *A*_3_ is a food company; (4)  *A*_4_ is an auto company; and (5)  *A*_5_ is a TV company. The criteria considered here in selection of the five possible companies are the following: (1)  *I*_1_ is cost; (2)  *I*_2_ is net present value; and (3)  *I*_3_ is loss, whose weight vector *w* = (0.3100,0.3600,0.3300)^*T*^. The investment company evaluates the performance of these companies *A*_*i*_  (*i* = 1,2,…, 5) in 2009–2011 according to the criteria *I*_*j*_  (*j* = 1,2, 3) and constructs the decision matrices *R*(*t*_*k*_) = (*β*_*ij*_(*t*_*k*_))_*m*×*n*_  (*k* = 1,2, 3, here, *t*_1_ denotes “2009”, *t*_2_ denotes “2010”, *t*_3_ denotes “2011”) as listed in Tables 2, 3, and 4 (unit: ten thousands RMB). In the decision matrices *R*(*t*_*k*_) = (*β*_*ij*_(*t*_*k*_))_*m*×*n*_, all the criterion values are expressed in log-normal distributions *β*_*ij*_(*t*_*k*_) = log⁡−*N*(*μ*_*ij*_(*t*_*k*_), (*σ*_*ij*_(*t*_*k*_))^2^), where *μ*_*ij*_(*t*_*k*_) and (*σ*_*ij*_(*t*_*k*_))^2^ can be estimated by using statistic methods (*i* = 1,2,…, 5, *j* = 1,2, 3, *k* = 1,2, 3).

To get the best company, the following steps are involved.

**Step 1**

Suppose that the relative average age *τ* = 0.2 by taking advice from the decision makers; then we use ((M-1)) to construct the optimization model and obtain time weight vector *λ*(*t*) = (0.0819,0.2363,0.6818)^*T*^.

**Step 2**

Utilize (16) to aggregate all the log-normal distribution decision matrices *R*(*t*_*k*_) = (*β*_*ij*_(*t*_*k*_))_5×3_ (*k* = 1,2, 3) into the overall log-normal distribution decision matrix *R* = (*β*_*ij*_)_5×3_ (Table 5).

**Step 3**

Utilize (18), (19), and (20) to normalize the decision matrix *R* = (*β*_*ij*_)_5×3_ into the corresponding decision matrix $\tilde{R}={\left({\tilde{\beta }}_{ij}\right)}_{5\times 3}$ (Table 6). Note that the criterion *I*_2_ is benefit criterion, and the criteria *I*_1_ and *I*_3_ are cost criteria.

**Step 4**

Utilize (21) to aggregate the overall criterion values ${\tilde{\beta }}_{ij}$ in the *i*th column of the normalized decision matrix $\tilde{R}={\left({\tilde{\beta }}_{ij}\right)}_{m\times n}$ and derive the complex overall values ${\tilde{\beta }}_{i}$ of the alternatives *A*_*i*_  (*i* = 1,2,…, 5):

(23) $$\begin{array}{c} {\tilde{\beta }}_{1}=\log-N\left(0.9146,0.0113^{2}\right),\\ {\tilde{\beta }}_{2}=\log-N\left(0.9067,0.0125^{2}\right),\\ {\tilde{\beta }}_{3}=\log-N\left(0.9285,0.0129^{2}\right),\\ {\tilde{\beta }}_{4}=\log-N\left(0.8550,0.0138^{2}\right),\\ {\tilde{\beta }}_{5}=\log-N\left(0.8645,0.0143^{2}\right). \end{array}$$

**Step 5**

Use (3) to calculate the expected values $\mu_{\log}({\tilde{\beta }}_{i})\;\;(i=1,2,\ldots ,5)$:

(24) $$\begin{array}{c} \mu_{\log}\left({\tilde{\beta }}_{1}\right)=2.4959,\;\;\mu_{\log}\left({\tilde{\beta }}_{2}\right)=2.4763,\\ \mu_{\log}\left({\tilde{\beta }}_{3}\right)=2.5309,\;\;\mu_{\log}\left({\tilde{\beta }}_{4}\right)=2.3516,\\ \mu_{\log}\left({\tilde{\beta }}_{5}\right)=2.3741. \end{array}$$

Thus,

(25) $$\begin{array}{c} \mu_{\log}\left({\tilde{\beta }}_{3}\right)>\mu_{\log}\left({\tilde{\beta }}_{1}\right)>\mu_{\log}\left({\tilde{\beta }}_{2}\right)>\mu_{\log}\left({\tilde{\beta }}_{5}\right)>\mu_{\log}\left({\tilde{\beta }}_{4}\right). \end{array}$$

**Step 6**

Use Definition 2 to rank all the alternatives *A*_*i*_  (*i* = 1,2,…, 5): *A*_3_≻*A*_1_≻*A*_2_≻*A*_5_≻*A*_4_. Therefore, the best alternative (company) is *A*_3_.

## 6. Conclusions

In this paper, we have proposed two new geometric aggregation operators, such as the LNDWG operator and the DLNDWG operator. Both operators can be used to aggregate the log-normally distributed random variables, can avoid losing the original decision information, and thus ensure the veracity and rationality of the aggregated results. But weights represent different aspects in both the LNDWG operator and the DLNDWG operator. The weights of the LNDWG operator only reflect the importance degrees of the given log-normal distributions themselves, whereas the weights of the DLNDWG operator only reflect the importance degrees of different periods. Thus, the LNDWG operator is a time independent operator, and because of taking time into account in the aggregation process, the DLNDWG operator is a time-dependent operator. The weights associated with the DLNDWG operator can be given by decision maker(s) or can be obtained by using the existing methods, but we have proposed another method by using the entropy model of Shannon. We have also developed an approach to dynamic SMCDM, in which the criterion values take the form of log-normally distributed random variables, and the argument information is given at different periods. This method has been detailedly illustrated with a practical example. This paper enriches and develops aggregation operator theory and SMCDM theory, and it can be widely applied in medical dynamic diagnosis, personnel dynamic examination, military system efficiency dynamic evaluation, and other related decision making fields. 

## Figures and Tables

**Table 1:** 0.1–0.9 scale for the relative average age *τ*.

*τ*	Implication
0.1	Paying more attention to recent data
0.3	Paying much attention to recent data
0.5	Paying the same attention to every period
0.7	Paying much attention to distant data
0.9	Paying more attention to distant data
0.2, 0.4, 0.6, 0.8	Intermediate values between adjacent scale values

**Table 2:** Decision matrix *R*(*t*_1_).

	*I*_1_	*I*_2_	*I*_3_
*A*_1_	log-*N *(385,9.2^2^)	log-*N *(259,7.9^2^)	log-*N *(139,5.6^2^)
*A*_2_	log-*N *(392,10.1^2^)	log-*N *(266,8.5^2^)	log-*N *(136,6.1^2^)
*A*_3_	log-*N *(358,8.9^2^)	log-*N *(253,6.8^2^)	log-*N *(130,6.8^2^)
*A*_4_	log-*N *(468,10.9^2^)	log-*N *(317,7.5^2^)	log-*N *(166,7.2^2^)
*A*_5_	log-*N *(451,9.6^2^)	log-*N *(303,6.9^2^)	log-*N *(159,7.5^2^)

**Table 3:** Decision matrix *R*(*t*_2_).

	*I*_1_	*I*_2_	*I*_3_
*A*_1_	log-*N *(371,9.6^2^)	log-*N *(251,7.6^2^)	log-*N *(134,5.5^2^)
*A*_2_	log-*N *(385,10.2^2^)	log-*N *(269,9.3^2^)	log-*N *(138,6.1^2^)
*A*_3_	log-*N *(359,9.3^2^)	log-*N *(253,8.6^2^)	log-*N *(135,6.5^2^)
*A*_4_	log-*N *(463,10.9^2^)	log-*N *(319,9.1^2^)	log-*N *(169,7.5^2^)
*A*_5_	log-*N *(455,9.7^2^)	log-*N *(319,8.9^2^)	log-*N *(155,8.6^2^)

**Table 4:** Decision matrix *R*(*t*_3_).

	*I*_1_	*I*_2_	*I*_3_
*A*_1_	log-*N *(369,9.2^2^)	log-*N *(255,7.9^2^)	log-*N *(131,5.7^2^)
*A*_2_	log-*N *(391,9.8^2^)	log-*N *(269,9.2^2^)	log-*N *(136,6.1^2^)
*A*_3_	log-*N *(351,10.6^2^)	log-*N *(257,8.6^2^)	log-*N *(133,6.7^2^)
*A*_4_	log-*N *(467,11.1^2^)	log-*N *(316,9.3^2^)	log-*N *(168,7.1^2^)
*A*_5_	log-*N *(469,11.7^2^)	log-*N *(306,8.8^2^)	log-*N *(158,7.6^2^)

**Table 5:** Overall decision matrix *R*.

	*I*_1_	*I*_2_	*I*_3_
*A*_1_	log-*N *(370.7830,6.7126^2^)	log-*N *(254.3824,5.7145^2^)	log-*N *(132.3641,4.1234^2^)
*A*_2_	log-*N *(389.6641,7.1511^2^)	log-*N *(268.7543,6.6827^2^)	log-*N *(136.4726,4.4299^2^)
*A*_3_	log-*N *(353.4637,7.5889^2^)	log-*N *(255.7272,6.2306^2^)	log-*N *(133.2269,4.8514^2^)
*A*_4_	log-*N *(466.1367,8.0440^2^)	log-*N *(316.7908,6.7236^2^)	log-*N *(168.0725,5.1886^2^)
*A*_5_	log-*N *(464.2176,8.3370^2^)	log-*N *(308.8262,6.3828^2^)	log-*N *(157.3730,5.5997^2^)

**Table 6:** Normalized decision matrix $\tilde{R}$.

	*I*_1_	*I*_2_	*I*_3_
*A*_1_	log-*N *(0.9533,0.0144^2^)	log-*N *(0.8030,0.0180^2^)	log-*N *(1.0000,0.0245^2^)
*A*_2_	log-*N *(0.9071,0.0153^2^)	log-*N *(0.8484,0.0211^2^)	log-*N *(0.9699,0.0264^2^)
*A*_3_	log-*N *(1.0000,0.0163^2^)	log-*N *(0.8072,0.0197^2^)	log-*N *(0.9935,0.0289^2^)
*A*_4_	log-*N *(0.7583,0.0173^2^)	log-*N *(1.0000,0.0212^2^)	log-*N *(0.7875,0.0309^2^)
*A*_5_	log-*N *(0.7614,0.0179^2^)	log-*N *(0.9749,0.0201^2^)	log-*N *(0.8411,0.0333^2^)
